# Effectiveness and safety of acupuncture and moxibustion for peripheral facial paralysis: A protocol for an overview of systematic reviews and meta-analysis

**DOI:** 10.1097/MD.0000000000022371

**Published:** 2020-09-18

**Authors:** Xingchen Zhou, Jun Xiong, Zhenhai Chi, Lunbin Lu, Jun Chen, Genhua Tang, Siyuan Zhu, Zhiying Zhong, Han Guo

**Affiliations:** a The Affiliated Hospital of Jiangxi University of Traditional Chinese Medicine; b Jiangxi University of Traditional Chinese Medicine, Nanchang, China.

**Keywords:** peripheral facial paralysis, acupuncture, moxibustion, AMSTAR-2, PRISMA, GRADEE, overview

## Abstract

**Background:**

Peripheral facial paralysis (PFP) seriously affects patients’ quality of life and work and even causes psychological problems such as anxiety and depression for them. Acupuncture (ACU) and moxibustion have been widely used to treat the disease with satisfactory results. Several systematic reviews and meta-analyses have reported the effectiveness of acupuncture for patients with PFP. However, the evidence has not been systematically synthesized. This overview aims to synthesize and assess the reliability of evidence generated from these systematic reviews (SRs) and meta-analyses of ACU and moxibustion for PFP.

**Methods:**

We will make a comprehensive retrieval in 9 databases as following: (1) Embase; (2) Cochrane Library; (3) Pubmed; (4) Chinese databases SinoMed (previously called the Chinese Biomedical Database); (5) Chinese National Knowledge Infrastructure (CNKI); (6) Chinese Scientific Journals Database (VIP); (7) Wanfang Data (WF). The time is limited from the construction of the library to August 2020. We will use the Assessment of Multiple Systematic Reviews-2 (AMSTAR-2) tool to evaluate methodological quality. Preferred Reporting Items for Systematic Reviews and Meta-analysis Protocols (PRISMA-P) will be used in the report checklist to assess the quality of reports in the study. The Grading of the Classification of Recommendations, Evaluation, Development and Evaluation (GRADE) will be used to evaluate the included SRs and meta-analysis. Our reviewers will conduct systematic reviews, qualification evaluation, data extraction, methodological quality and evidence quality screening in pairs. The outcomes of interest include: the effective rate, the House-Brackmann (H-B) score, cure rate, and side effects. Or any other scale used to assess the level of illness. The evidence will be synthesized where appropriate based on patient subgroups and outcomes.

**Results:**

The results will be published in a peer-reviewed journal.

**Conclusion:**

This overview will provide comprehensive evidence of ACU and moxibustion for patients with PFP.

**Trial registration number:**

INPLASY202080016.

## 1 Introduction

Peripheral facial paralysis is a common clinical disease and frequently-occurring disease. From the perspective of social psychology, this disease will produce anxiety and depression, which will seriously affect patients’ life and work.^[1–3]^ The clinical manifestations mainly include shallowing or disappearing off the frontal lines on the affected side, inability to move the expression muscles, incomplete eyelid closure, shallowing or disappearing of the nasolabial fold, crooked mouth corners to the good side, poor whistling, and bulging cheeks.^[4]^ According to reports, the incidence of different populations is about 11.5 to 53.3 per 100,000 people.^[5]^ One out of every 60 patients will be affected in their whole life.^[6]^ Studies have shown that 71% of BFP patients have restored normal facial function, while 29% of patients have symptoms of hemifacial weakness, and even more than half of the patients are disfigured.^[7]^ Studies have shown that PFP may also increase the occurrence of cardiovascular and cerebrovascular diseases.^[8,9]^ Therefore, it is necessary to study the labor loss caused by PFP to improve patients’ physical and mental health.

Modern medicine believes that the occurrence of PFP is often directly related to viral infection. The most common cause of PFP is Bell's palsy, which Related to specifically herpes simplex virus type I.^[10]^ Ramsay Hunt syndrome is the second most common cause of atraumatic peripheral facial paralysis.^[11]^ Other causes of acquired PFP are less common, including the Ramsay Hunt syndrome, Lyme disease, etc.^[12]^

Western medicine commonly used in the treatment of PFP include corticosteroid, antiviral, neurotrophic, vitamin B, and surgery.^[13–15]^ These treatments can indeed play a pivotal effect, but the side effects of the drugs are daunting. Corticosteroids can induce or aggravate peptic ulcers, cause malignant hypertension, and affect the blood supply to the liver and kidneys.^[16]^ Antiviral drugs can cause digestive symptoms such as nausea, vomiting, and diarrhea.^[17]^ Nutrient neurological drugs are usually only used as alternative and complementary therapies, and cannot play a leading therapeutic effect. Surgical therapy occasionally presents dangerous risks.^[18]^ These risk factors prompt patients to seek safer and more effective alternative therapies, such as AVU and moxibustion, which are considered the best options by the general public.

PFP belongs to “skewed mouth and eyes” in Traditional Chinese Medicine (TCM).^[19]^ Based on the TCM, the pathogenesis is the foot Yangming meridian; the foot sun meridian and the hand sun meridian are out of balance.^[20,21]^ And The veins’ emptiness is the internal cause, and the invasion of thieves is the external cause. The internal and external factors interact with each other to cause the disease. That is, the patient is usually physically weak; lack of righteousness and external health is not substantial. The external evil invades the facial muscles, causing the facial Qi and blood to run out of balance, and the muscles are dysfunctional.^[22]^

ACU and moxibustion are appropriate TCM therapies, and the treatment of PFP has a long history and long-term clinical practice. A large number of literature reports believe that these therapies have unique clinical treatment methods for PFP.^[23,24]^ Studies have found that ACU and moxibustion can regulate the relationship between endothelin secreted by vascular endothelial cells and microcirculation, thereby regulating vasoconstriction or hemodynamics, and ultimately treating PFP.^[25]^ Besides, these therapies can also speed up the left facial nerve's conduction speed, promote the expression of nerve growth factor protein, brain-derived neurotrophic factor protein, BDNF mRNA, and its protein in the facial nerve nucleus, thereby enhancing facial nutrition. Nerves and promoting the growth of facial nerve axons play a role in the regeneration and repair of nerves after injury.^[26]^

Many randomized controlled trials (RCTs) have confirmed the efficacy of ACU and moxibustion in PFP treatment.^[27,28]^ Many meta-analyses also show that ACU and moxibustion treatment has specific benefits for PFP patients.^[24,29]^ However, there has not been a rigorously designed overview to evaluate the systematic evaluation of PFP by ACU and moxibustion. Therefore, this study evaluates and summarizes the clinical research literature of ACU and moxibustion in the treatment of peripheral facial paralysis at home and abroad to provide evidence-based medicine for clinical practice.

## 2 Methods

### 2.1 Study registration

This protocol was designed in accordance with the methodological guidelines for overviews provided by the Cochrane Handbook for Systematic Reviews of Interventions.^[30]^ It is registered on the International Prospective Register of Systematic Reviews (registration number INPLASY202080016; https://inplasy.com/inplasy-2020-8-0016/).

### 2.2 Inclusion and exclusion criteria

PICOS will be applied, including Population, Intervention, Comparison, Outcome, and Study.

#### 2.2.1 Type of study

It only includes systematic reviews and meta-analysis of randomized controlled trials (RCT) on ACU and moxibustion in PFP patients published in English and Chinese.

#### 2.2.2 Type of participants

It will include a systematic review of people diagnosed with PFP. Regardless of gender, race, occupation, education, nationality, etiology, and severity, all PFP study participants of all ages can be included.

#### 2.2.3 Type of interventions

ACU treatments include moxibustion, catgut embedding, electro-acupuncture, transcutaneous electrical acupoint stimulation, auricular acupuncture, scalp acupuncture, warm needling, manual acupuncture, acupoint injection, regardless of needling techniques and stimulation method.

#### 2.2.4 Type of comparator (s)/control

The control group's treatment is not limited, including no treatment, placebo, or any control considered for comparison in a single systematic review.

#### 2.2.5 Types of outcome measurements

##### 2.2.5.1 Primary outcomes

The total effective rate of ACU and moxibustion to treat PFP will be the primary outcome.

##### 2.2.5.2 Secondary outcomes

Secondary outcomes mainly include the following aspects:

1. House-Brackmann (H-B) score.
2. Cure rate.
3. Advert events.

#### 2.2.6 Study design

SRs containing more than one RCT were included. There is no systematic reviews, no separate summary of RCT data, and abstracts without sufficient data will be excluded.

### 2.3 Search methods for identification of studies

We searched 3 foreign electronic databases (Cochrane Library, Embase, Pubmed) and 4 Chinese electronic databases (China National Knowledge Infrastructure (CNKI), WangFang Database, Chinese Biomedical Literature Database (CBM) and Chinese Scientific Journal Database (VIP) to collect potential systematic reviews (SRs) from their inceptions to August 2020. The language of publication is limited to Chinese or English. The following search terms will be used: peripheral facial paralysis, bell palsy, facial paralysis, facial nerve, facial nerve disease, facial nerve paralysis, moxibustion, thunder fire miraculous moxa roll, thunder fire moxibustion, taiyi miraculous moxa roll, suspended moxibustion, mild moxibustion, needle warming moxibustion, systematic review, meta-analysis, et al. A draft search strategy using Pubmed, one of the planned electronic databases to be searched, is presented in Table 1.

**Table 1:**
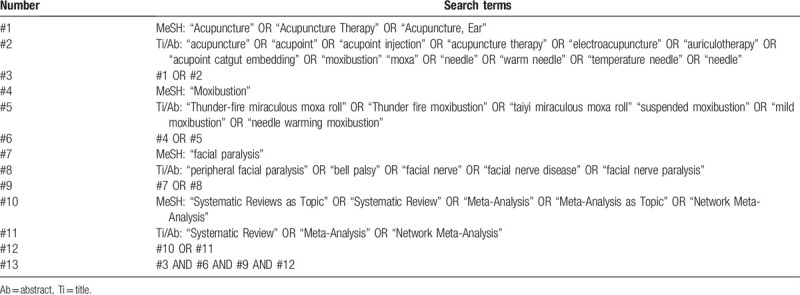
The search strategy for Pubmed.

### 2.4 Studies selection

**Figure 1:**
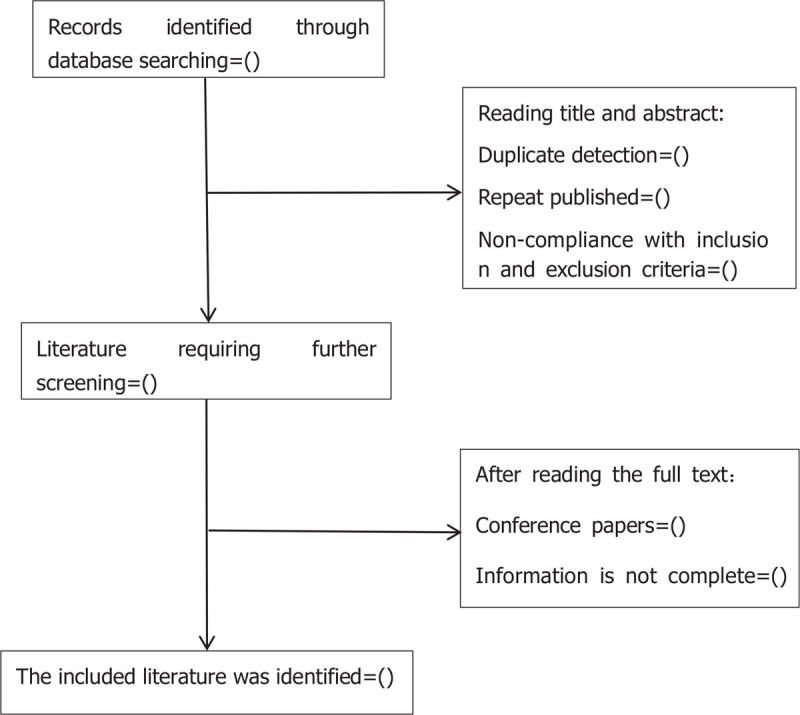
Flowchart of literature selection.

Studies will be identified using NoteExpress 3.2.0. After the initial removal of duplicate studies, 2 reviewers (ZHC and LBL) will independently screen titles and abstracts based on the eligibility criteria. Full-text studies will be retrieved for all potentially includable SRs or SR protocols. If studies contain insufficient information to make a decision about eligibility, QSX will try to contact authors of the original reports to obtain further details. During the procedure, disagreements will be resolved by discussion or consensus with the third reviewer (JC). Study selection will be performed in accordance with the PRISMA flowchart (Fig. 1).

### 2.5 Data extraction

Two researchers (SYZ and GHT) extracted literature information based on inclusion and exclusion criteria, including the following:

(1) Study characteristics: author, year, study design, sample size and follow-up time;
(2) Patient characteristics: age, sex, and type of SP;
(3) Intervention: intervention measures in the experimental group, intervention measures in the control group);
(4) Outcome of the study: Two researchers (ZYZ and HG) cross-checked the extraction results of the extracted documents. If there is any difference, you should consult a third party (ZHC) to resolve.

#### 2.5.1 Evaluate the methodological quality of included studies

Two reviewers (XCZ and HG) will use the Assessment of Multiple Systematic Reviews 2 (AMSTAR-2) measurement tool to independently assess each SRs methodological quality that meets the inclusion criteria.^[29]^ This is most commonly used to assess the quality of systematic reviews included in overviews. AMSTAR-2 is an update of AMSTAR, which can be used to appraise SRs of both randomized and non-randomized controlled trials. AMSTAR-2 includes 16 items, with each of the 16 criteria given a rating of “yes” (definitely done), “no” (definitely not done), “cannot report” (unclear if completed), or “not applicable” based on the information provided by the systematic review, when the standards are met, the evaluator will evaluate the evaluation. Disagreements will be resolved through discussions between them and arbitrated by the third general author (GHT) if necessary.

### 2.6 Evaluation of the reporting quality of the included studies

The 2 authors of the overview (LBL and JC) will independently evaluate the reports’ quality in each review to assess whether they meet the criteria specified in Preferred Reporting Items for Systematic Reviews and Meta-analysis Protocols (PRISMA-P).^[30]^ If there are any differences, they will be resolved through discussion between them and arbitrated by the third general author (XCZ) if necessary.

### 2.7 Evaluation of the evidence quality of the included studies

The quality of evidence of the included SRs was assessed by the Grading of Recommendations Assessment, Development and Evaluation (GRADE) approach.^[31]^ This tool aims to assess the quality of evidence for each outcome indicator in the study. The 2 authors (SYZ and ZYZ) will independently evaluate the evidence of the results and should describe in detail the degradation or upgrade factors that affect the quality of the evidence to ensure the reliability and transparency of the results. Any disagreements will be resolved through discussion by 2 authors. The overall quality of evidence was judged as “high,” “moderate,” “low,” or “very low.”

### 2.8 Dealing with lost data

If there is no specific data or insufficient data in the published SRs, the author will be contacted by email or phone to provide the necessary information. If we cannot obtain enough data, the data will be discarded. The analysis will be based on the available data and the potential impact of missing data will be discussed.

### 2.9 Synthesis of data

Before data synthesis, the included SRs and meta-analysis should be considered. For different situations, different measures will be taken for overlapping basic research: whether the basic research completely overlaps, the comment with the highest quality will be selected. If the main research partially overlaps, when the lower quality reviews include more than one-third of the new research. If the basic research does not overlap, the 2 comments will remain. The quality of the review will be fully assessed Use ROBIS and AMSTAR-2. Besides, RevMan5.3.5 software will be used to calculate the standardized effect. The random-effects model (*I*^2^ ≥ 50%) or fixed-effects model (*I*^2^ < 50%) will be selected according to the heterogeneity levels of the included SRs and meta-analyses. If the *I*^2^ value is higher than 75%, the clinical or methodological heterogeneity will be explored through discussion with the review team. When the meta-analysis is not possible, a narrative analysis will be performed. Indirect comparisons of different ACU and moxibustion therapies will also be conducted using relative effectiveness outcomes including relative sensitivity and relative specificity.

## 3 Discussion

PFP is a disease that frequently occurs at any age. It not only seriously affects the work and life of patients, but also produces depression and anxiety. ACU and moxibustion as an effective technique of TCM, has been accepted for PFP in China. However, due to the lack of rigorous review evidence for ACU and moxibustion treatment, clinicians cannot choose the best method. As a result, patients with peripheral facial paralysis are prone to delays. Therefore, The results of this overview will provide real and reliable research evidence for the treatment of peripheral facial paralysis with acupuncture and moxibustion.

The study also has some defects as follows: low quality of original researches, the possible occurrence of false positive or false negative results, various duration of disease, different dosage, and frequency of intervention, language restriction, etc. All of these will lead to some bias and influence the results of evaluation results, ultimately affecting this study's reliability.

## Author contributions

All authors have read and approved the publication of the protocol.

**Conceptualization:** Xingchen Zhou, Jun Xiong.

**Data curation:** Xingchen Zhou, Zhenhai Chi, Lunbin Lu, Jun Chen, Genhua Tang, Siyuan Zhu, Zhiying Zhong, Han Guo.

**Formal analysis:** Xingchen Zhou, Zhenhai Chi.

**Investigation:** Xingchen Zhou, Jun Xiong, Zhenhai Chi.

**Methodology:** Xingchen Zhou, Lunbin Lu, Jun Chen, Genhua Tang.

**Software:** Zhenhai Chi, Siyuan Zhu.

**Supervision:** Jun Xiong, Zhenhai Chi.

**Writing – original draft:** Xingchen Zhou, Jun Xiong, Lunbin Lu, Han Guo.

**Writing – review & editing:** Jun Xiong, Zhenhai Chi, Lunbin Lu, Jun Chen, Genhua Tang.

## Abbreviations

Abbreviations: ACU = acupuncture, AMSTAR-2 = Assessment of Multiple Systematic Reviews-2, GRADE = Grading of Recommendations, Assessment, Development, and Evaluation, H-B = the House-Brackmann score, PFP = peripheral facial paralysis, PRISMA = Preferred Reporting Items for Systematic Reviews and Meta-Analyses, PRISMA-P = Preferred Reporting Items for Systematic Reviews and Meta-analysis Protocols, RCTs = randomized controlled trials, SRs = systematic reviews, TCM = Traditional Chinese Medicine.
